# Donor Fractions of Cell-Free DNA Are Elevated During CLAD But Not During Infectious Complications After Lung Transplantation

**DOI:** 10.3389/ti.2024.12772

**Published:** 2024-07-24

**Authors:** Mirza Novo, Rickard Nordén, Johan Westin, Göran Dellgren, Jens Böhmer, Anne Ricksten, Jesper M. Magnusson

**Affiliations:** ^1^ Department of Respiratory Medicine, Institute of Medicine, Sahlgrenska Academy, University of Gothenburg, Gothenburg, Sweden; ^2^ Department of Clinical Microbiology, Sahlgrenska University Hospital, Gothenburg, Sweden; ^3^ Department of Infectious Diseases, Institute of Biomedicine, Sahlgrenska Academy, University of Gothenburg, Gothenburg, Sweden; ^4^ Transplant Institute, Sahlgrenska University Hospital, Gothenburg, Sweden; ^5^ Department of Cardiothoracic Surgery, Institute of Clinical Sciences, Sahlgrenska Academy at the University of Gothenburg, Gothenburg, Sweden; ^6^ Pediatric Heart Center, Queen Silvia Children’s Hospital, Sahlgrenska University Hospital, Gothenburg, Sweden; ^7^ Department of Pediatrics, Institute of Clinical Sciences, Sahlgrenska Academy, University of Gothenburg, Gothenburg, Sweden; ^8^ Department of Pediatrics, Clinic Frankfurt-Höchst, Frankfurt, Germany; ^9^ Department of Clinical Genetics and Genomics, Sahlgrenska Academy, University of Gothenbururg, Gothenburg, Sweden

**Keywords:** lung transplantation, allograft dysfunction, biomarker, cell-free DNA, droplet digital PCR

## Abstract

During the last few years, cell-free DNA (cfDNA) has emerged as a possible non-invasive biomarker for prediction of complications after lung transplantation. We previously published a proof-of-concept study using a digital droplet polymerase chain reaction (ddPCR)-based method for detection of cfDNA. In the current study, we aimed to further evaluate the potential clinical usefulness of detecting chronic lung allograft dysfunction (CLAD) using three different ddPCR applications measuring and calculating the donor fraction (DF) of cfDNA as well as one method using the absolute amount of donor-derived cfDNA. We analyzed 246 serum samples collected from 26 lung transplant recipients. Nine of the patients had ongoing CLAD at some point during follow-up. All four methods showed statistically significant elevation of the measured variable in the CLAD samples compared to the non-CLAD samples. The results support the use of ddPCR-detected cfDNA as a potential biomarker for prediction of CLAD. These findings need to be validated in a subsequent prospective study.

## Graphical Abstract

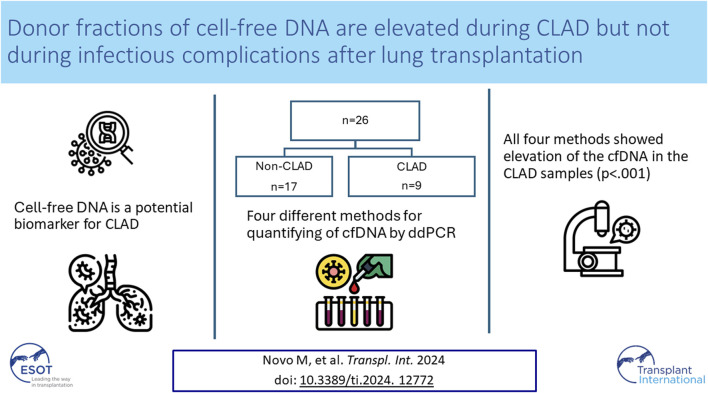

## Introduction

Lung transplantation is a lifesaving treatment for patients with irreversible nonmalignant lung disease. During the last 30 years, approximately 70,000 adult lung transplant procedures have been performed worldwide [1]. Despite advances in organ procurement, improved surgical techniques and perioperative care, lung transplant patients have the shortest survival of all the major organ transplantation [2, 3] with a current median survival of 6.7 years [4].

The main limiting factor for survival is the high-rate development of chronic lung allograft dysfunction (CLAD) [4]. CLAD is currently defined as an irreversible decline of forced expiratory volume 1 s (FEV1) to ≤80% of a baseline FEV1.

Although several risk factors such as primary graft dysfunction [5], infections e.g., bacterial, viral and fungal [6], esophageal reflux [7], anti-HLA antibodies [8], acute cellular rejection [9] and choice of immunosuppression [10] have been proposed, the cause of CLAD remains elusive. Furthermore, the occurrence of risk factors does not adequately predict CLAD development. Several biomarkers associated with CLAD have been proposed, but their application in clinical practice has been limited due to insufficient specificity and sensitivity, as well as failure to detect early-stage disease [11, 12]. A reliable biomarker for isolated allograft damage would facilitate early detection of CLAD in a clinical setting and thus enable early therapeutic intervention [11], which would likely improve outcomes after LTx.

Cell-free DNA (cfDNA) can be released from injured cells into the bloodstream and detected in samples from bronchoalveolar lavage [13], urine, cerebrospinal fluid [14], as well as plasma and serum [15]. Quantification of cfDNA has been proven as a potential biomarker for the prediction of various diseases, including malignancy [16, 17], myocardial infarction [18], sepsis [19] and traumatic injuries [20]. After transplantation with a donated solid organ, two distinctly different sets of cfDNA may exist within the same individual, either donor-derived cfDNA (dd-cfDNA) or recipient-derived cfDNA (rd-cfDNA). Quantification of dd-cfDNA in transplant recipients has been shown to be useful for prediction of acute rejection in lung [21, 22], kidney [23], liver [24] and heart [25] transplantation.

We previously published a proof-of-concept study using droplet digital polymerase chain reaction (ddPCR) to quantify dd-cfDNA and rd-cfDNA in peripheral blood [26], showing potential to differentiate between CLAD and non-CLAD samples. Previously published methods, using various sequencing techniques, have solely reported the ratio between the two sources of cfDNA, referred to as donor fraction (DF) [21, 27, 28], which can still be calculated using our methodology [26]. The methodology also makes it possible to present donor and recipient cfDNA separately with quantification of the respective type of cfDNA [29, 30], which have already been proven in kidney [31] and liver transplant [32]. Moreover, ddPCR is practical in a clinical setting due to its fast turnaround time [31] and has the advantage to be both very sensitive and cost-efficient when compared to next-generation sequencing (NGS) based approaches [33, 34]. There are known variations in the total levels of cell-free DNA, both in pathological and physiological conditions [35, 36]. Donor fraction alone does not account for these fluctuations, and studies have shown absolute levels of dd-cfDNA perform better than DF after kidney transplantation [31, 37]. In this study, where we retrospectively used available samples from a prospective study, we found that the quantity and relative proportion of dd-cfDNA reflected several clinical effects, e.g., allograft damage. Samples were collected according to a fixed protocol. Samples collected 1 month after transplantation were consistently elevated, potentially confounding overall measurements. We also observed that any systemic affliction of the donor was associated with elevations of both the rd-cfDNA and dd-cfDNA, which might lead to a low DF despite CLAD.

This study aimed to evaluate our method further as a biomarker for CLAD, testing faster ways to process the PCR results and the impact of simplification on precision. In addition, the results in the proof-of-concept study also suggested that the absolute amount of dd-cfDNA could possibly be correlated to CLAD which was also evaluated further in the current study.

## Materials and Methods

### Patients and Study Design

Patients from a previously published cohort of patients undergoing lung transplantation between 2009 and 2011 at Sahlgrenska University Hospital were included [38, 39]. This cohort recorded and collected clinical status and samples at scheduled outpatient visits after LTx at 1, 2, 3, 4.5, 6, 9, 12, 18, 24, and 36 months. Furthermore, samples were also collected at every extra outpatient visit during this period. From this pool of previously collected serum samples, patients were selected based on serum availability. Patients with at least five samples from five separate time-points remaining were identified and included. Previously thawed samples were excluded. No samples from the proof-of-concept study [26] were used in the current study.

Induction therapy consisted of rabbit antithymocyte globulin, which was given for 1 to 3 consecutive days together with methylprednisolone IV. Post-transplantation immunosuppression included prednisone, 0.3 mg/kg/day and mycophenolate mofetil, 2 g/d. The patients then received either oral cyclosporine (CSA) (1-2 mg/kg) adjusted to maintain a serum level of 300–350 ng/mL or tacrolimus (TAC), 0.075 mg/kg given orally divided in 2 doses daily adjusted to maintain a serum level of 14–16 ng/mL. The dosage of immunosuppression was gradually lowered during follow up. Further changes in immunosuppressive therapy were based on clinical presentation [38]. For some patients, viral airway infections prompted a transient 1-to-3-week elevation of prednisone to approximately 0.3 mg/kg, according to local clinical deliberations. No other adjustments to base immunosuppression were made based on clinical events for any of the patients.

Respiratory viral agents were screened for at all outpatient visits. Bronchiolar lavage samples at 1, 3, and 12 months and for-cause were cultured for bacterial and fungal agents and airway viral agents. A previously described multiplex PCR, able to detect 17 viral agents [40], was used for respiratory viral agents. PCR-quantification was used for cytomegalovirus (CMV) and Epstein-Barr virus detection in all samples. All samples were processed at the hospital’s routine clinical microbiological laboratory. If a positive sample constituted a clinically relevant infection, it was evaluated by an experienced clinician. Data regarding patient characteristics and clinical events was retrieved from electronic patient case report forms.

All serum samples were centrifuged at 3,000 × g after collection and aliquoted before frozen at −80°C within 24 h after sampling. The laboratory staff was blinded to all clinical and patient‐related data. Serum samples were identified by serial numbers only during analysis and data management.

CLAD was defined as an irreversible loss of >20% of baseline FEV1, confirmed with at least two spirometries at least 3 weeks apart, where all other possible differential diagnoses such as infections, acute rejection, airway stenosis and antibody-mediated rejection had been excluded. At CLAD diagnosis, all patients with CLAD had been on Azithromycin 250 mg three times a week for more than 3 months at time of diagnosis. The CLAD diagnosis could be possible, probable or definite based on the time since initial loss of function (<3 weeks, 3 weeks-3 months or >3 months) without restitution or discovery of other more likely differential diagnoses. A loss of >10% of total lung capacity and restrictive allograft syndrome (RAS)-like opacities indicates the subtype RAS. The samples collected at the time of CLAD diagnosis 3 months before and after CLAD diagnosis were denominated as CLAD.

### DNA Isolation and Genotyping

Whole blood samples were used for genotyping. Donor and recipient genomic DNA was extracted from EDTA‐blood preparations using the DNeasy Blood & Tissue Kit (Qiagen).

A panel of 35 highly polymorphic SNP (single‐nucleotide polymorphism) assay was used together with ddPCR (QX200 AutoDG Droplet digital PCR System, Bio-Rad) for genotyping and selection of informative assays to discriminate recipient DNA from donor DNA. Per recipient, 2-3 informative SNP assays were selected.

### cfDNA Isolation, Target‐Specific Preamplification and Analysis

Serum samples were used for longitudinal detection of cfDNA. cfDNA was extracted from 0.25 to 1.25 mL serum using the QIAamp^®^ Circulating Nucleic Acid Kit (Qiagen) according to the manufacturer’s protocol. Concentrations of cfDNA were quantified with the Qubit^®^ 3.0 Fluorometer (Thermo Fisher Scientific), fragment sizes were analyzed with the 4200 TapeStation (Agilent Technologies).

Absolute levels of donor and recipient cfDNA were quantified by ddPCR using one of the informative SNP assays. Calculations of copies were performed by Quant Soft (BioRad).

Target preamplification of cfDNA was performed using pooled primers for all 35 SNP [26]. The preamplified cfDNA was quantified by ddPCR using the informative SNP assays. The SNP assays were analyzed in triplicates, all experiments were included with no template controls. The copies generated by ddPCR for each allele at each SNP locus were calculated using Quanta Soft (Bio‐Rad). The mean value from triplicate assays was used to calculate the levels of dd‐cfDNA, rd‐cfDNA, and DF.

At least five samples per patient must be adequately analyzed for the patient to be included in the final analysis. Samples were excluded due to sample hemolysis, insufficient plasma yield, high technical error rate and failed droplet generation.

Sample results were categorized by baseline groups but also by fungal, viral, and bacterial infectious events as well as CLAD, depending on analysis. Samples without the analyzed property or event at the time of sampling were used as controls. Events where no samples were available were not included.

Four distinct methodologies to analyze the results from the ddPCR were applied. DF calculated from each pre-amplified SNP individually was labelled Method 1 (M1). DF calculated from the mean of all pre-amplified SNPs per event was labelled method 2 (M2). DF calculated from the first not pre-amplified SNP was labelled Method 3 (M3). Finally, using only the absolute value of dd-cfDNA quantified by the non-pre-amplified dd-PCR, was labelled Method 4 (M4). Means over all samples per individual were used for groupwise baseline comparisons. For infectious events and CLAD, comparisons were made between event and non-event samples.

### Statistics

Data were analyzed by SPSS for macOS v 29.0. Values were presented as median and interquartile range (IQR). Comparisons at the group level were performed using the Mann–Whitney U test. *p* < .05 was considered statistically significant. ROC (receiver operating characteristic) curve and AUC (area under the curve) were used to calculate evaluation metrics of the different aspects of ddPCR-based cfDNA.

## Results

**FIGURE 1:**
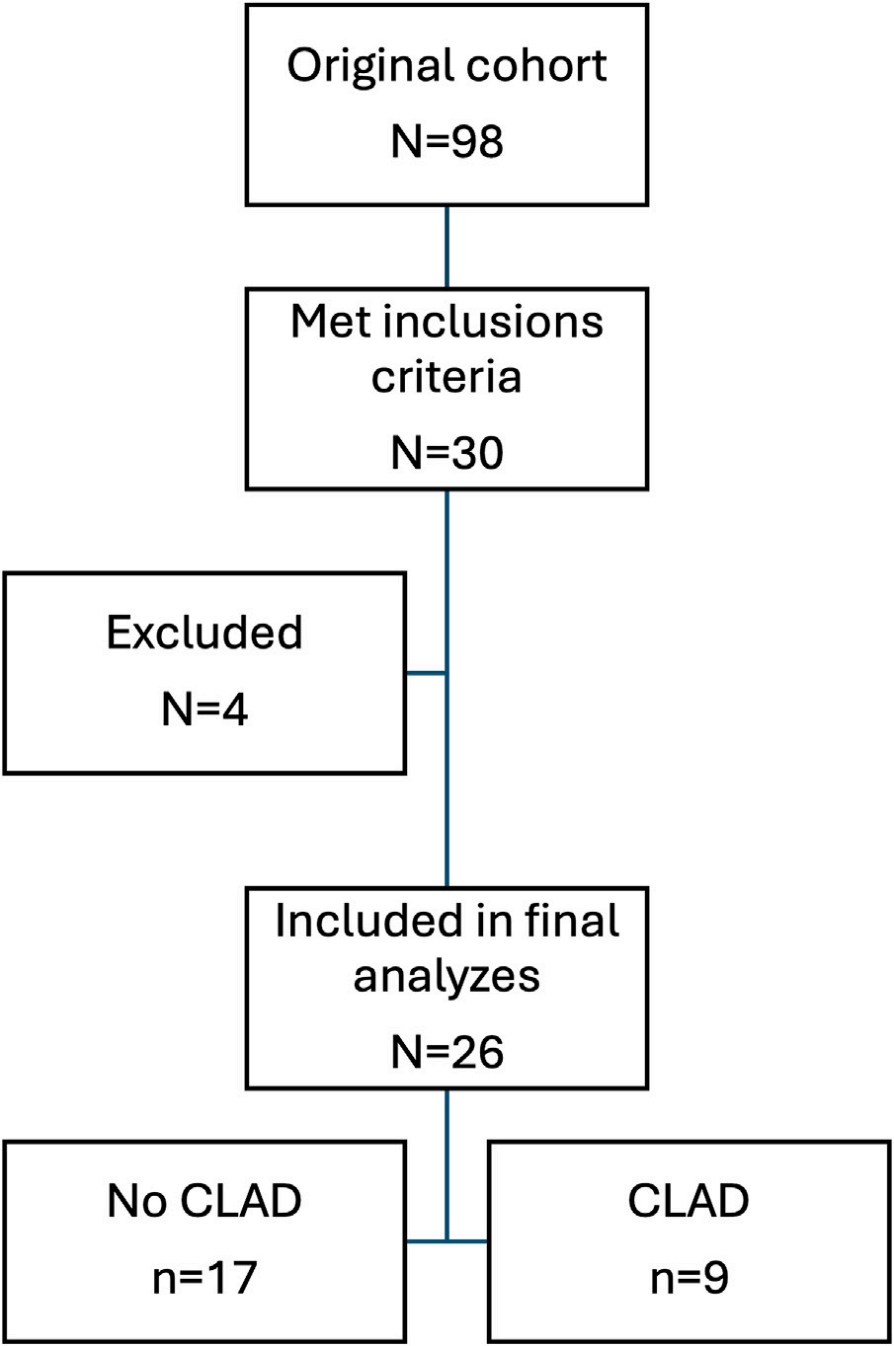
Flow chart showing patient selection.

Thirty patients matched the inclusion criteria in the biobank. Samples from four patients (two male and two female) were excluded. Two were excluded due to fewer actual samples in the biobank than indicated, and two were excluded because of the high rate of technical errors in one sample each or fewer actual samples to analyze in the biobank than indicated (Figure 1). The technical errors in both samples did not show any difference compared to other signals for the PCR due to a high level of background genomic DNA, suggesting it to be the result of pre-analytical factors. Furthermore, in one of the two samples, one of the instruments failed and was unable to read one of the tested SNPs, and there was not sufficient remaining volume to re-do the test.

Twenty-six patients (15 female and 11 male) were included in the final analysis, of which nine (36.4%) developed CLAD at some point during follow-up with a max of 16 CLAD samples. The median age at the time of transplantation was 51 (IQR 42–63) years. Most of the patients were transplanted because of pulmonary fibrosis – 46.2% and chronic obstructive pulmonary disease (COPD) – 30.8%. Bilateral lung transplants were the most common (69.2%) (Table 1).

**TABLE 1:** Baseline characteristics of the study population (N = 26).

Variable		
Sex	Female, n (%)	15 (57.7%)
	Male, n (%)	11 (42.3%)
Age at time of transplantation, years	Median (IQR)	51 (41–63)
BMI (kg/m^2^)	Median (IQR)	23.1 (19.7–21.7)
Indication for transplantation	Pulmonal fibrosis, n (%)	12 (46.2%)
	COPD, n (%)	8 (30.8%)
	Alpha-1 trypsin deficiency, n (%)	3 (11.5%)
	Other, n (%)	3 (11.5%)
Type of transplantation	Single, n (%)	8 (30.8%)
	Double, n (%)	18 (69.2%)
Mismatch	Cytomegalovirus, n (%)	6 (23.1%)
	Epstein-Barr virus, n (%)	1 (3.8%)
CLAD during follow-up	None, n (%)	17 (65.4%)
	CLAD, n (%)	9 (34.6%)

n, number; IQR, interquartile range; COPD, chronic obstructive pulmonary disease. Mismatch–seropositive donor and seronegative recipient. CLAD, chronic lung allograft dysfunction.

At the end of clinical follow-up, all the included CLAD patients had observed persistent graft dysfunction for more than 3 months and could, therefore, be defined as definite CLAD.

At baseline, we found no differences between patients who developed CLAD during follow-up and those who did not, nor any difference based on sex (Supplementary Table S1).

Females had significantly higher overall DF compared to males for M1 (*p* = 0.011), M2 (*p* = 0.036), M3 (*p* = 0.036), and M4 (*p* = 0.047). However, there were no differences in transplant type or CMV mismatch (Table 2).

**TABLE 2:** Characteristics of the study population regarding levels of DF calculated by four methods.

Variable	Median (IQR)	Variable	Median (IQR)	*p*-value
Sex				
Male		Female		
M 1	0.049 (0.030–0.120)	M 1	0.130 (0.070–0.200)	**0.011**
M 2	0.047 (0.030–0.122)	M 2	0.130 (0.061–0.202)	**0.036**
M 3	0.046 (0.018–0.161)	M 3	0.143 (0.095–0.344)	**0.036**
M 4	0.084 (0.037–0.288)	M 4	0.322 (0.143–1.204)	**0.047**
Type of transplantation				
Double		Single		
M 1	0.120 (0.040–0.180)	M 1	0.110 (0.040–0.150)	0.810
M 2	0.118 (0.04–0.168)	M 2	0.118 (0.040–0.180)	0.978
M 3	0.161 (0.053–0.229)	M 3	0.010 (0.022–0.195)	0.311
M 4	0.202 (0.046–1.04)	M 4	0.233 (0.077–0.390)	0.892
Mismatch CMV				
**Yes**		**No**		
M 1	0.080 (0.030–0.240)	M 1	0.110 (0.050–0.160)	0.930
M 2	0.082 (0.300–0.243)	M 2	0.120 (0.048–0.168)	0.790
M 3	0.107 (0.064–0.256)	M 3	0.118 (0.025–0.206)	0.882
M 4	0.305 (0.117–1.960)	M 4	0.214 (0.056–0.431)	0.295

M1 Method 1 DF calculated from each pre-amplified SNP, individually (n = 665).

M2 Method 2 DF calculated from mean of all pre-amplified SNPs, per event (n = 221).

M3 Method 3 DF Calculated from the first non-pre-amplified SNP (n = 198).

M4 Method 4 The absolute value of dd-cfDNA, quantified by the non-pre-amplified dd-PCR (n = 218).

Data are presented as median (Md) and interquartile range (IQR). Mismatch–seropositive donor and seronegative recipient. CMV, cytomegalovirus. The statistic calculations were done using Mann-Whitney U test. Significant *p*-values are highlighted in bold.

The analysis of the dynamics over time showed that the samples available at one-month post-transplantation had significantly higher levels of M1 (*p* < 0.001), M2 (*p* < 0.001), M3 (*p* = 0.005), and M4 (*p* = 0.007) compared to all subsequent samples (Supplementary Table S2). There were too few samples after the CLAD diagnosis to perform any meaningful analysis on post-CLAD dynamics. At the end of follow-up, only two patients had developed the RAS subtype (3 samples), and there were no significant differences in any of M1-M4 (*p* > 0.05). Further analyses were performed with one-month samples excluded and no subdivision of CLAD samples.

The analysis of individual events showed that viral, bacterial or fungal infection M1, M2, M3 showed no significant difference between samples at the event and samples without the event. However, for M4, the test results for viral (*p* = 0.034) and fungal (*p* = 0.021) were significantly elevated whilst there were no significant differences for samples with bacterial infections. The DF levels and the dd-cfDNA level respectively, for samples with CLAD were elevated compared to samples without CLAD for M1 (*p* < 0.001), M2 (*p* < 0.001), M3 (*p* < 0.001) and M4 (*p* < 0.001) (Table 3). Only two patients developed acute rejection (AR) at any time during follow-up and none of these events had matching samples. No patient had a CLAD sample with a simultaneous infection of any kind.

**TABLE 3:** Levels of donor fraction DF of cfDNA obtained by four different methods with regard to different infections and CLAD.

Method 1 DF calculated from each amplified SNP individually (n = 665)					
	n	No	n	Yes	*p*-value
		Median (IQR)		Median (IQR)	
Viral infection	382	0.060 (0.021–0.171)	283	0.052 (0.019–0.136)	0.118
Bacterial infection	611	0.060 (0.021–0.164)	54	0.038 (0.018–0.105)	0.125
Fungal infection	629	0.054 (0.021–0.154)	36	0.086 (0.035–0.157)	0.216
CLAD	616	0.050 (0.020–0.150)	49	0.120 (0.070–0.310)	**<0.001**

Method 2 DF calculated from mean of all amplified SNPs per event (n = 221)					
	n	**No**	n	**Yes**	*p*-value
		Median (IQR)		Median (IQR)	
Viral infection	126	0.063 (0.026–0.151)	95	0.051 (0.021–0.153)	0.462
Bacterial infection	203	0.062 (0.026–0.159)	18	0.045 (0.020–0.130)	0.415
Fungal infection	209	0.059 (0.024–0.151)	12	0.101 (0.048–0.156)	0.200
CLAD	205	0.051 (0.022–0.140)	16	0.222 (0.089–0.329)	**<0.001**

Method 3 DF calculated from the first non-pre-amplified SNP (n = 198)					
	n	No	n	Yes	*p*-value
		Median (IQR)		Median (IQR)	
Viral infection	117	0.059 (0.012–0.174)	81	0.087 (0.026–0.195)	0.177
Bacterial infection	181	0.070 (0.015–0.191)	17	0.061 (0.027–0.160)	0.981
Fungal infection	188	0.068 (0.015–0.182)	10	0.110 (0.049–0.269)	0.274
CLAD	183	0.059 (0.015–0.167)	15	0.388 (0.097–0.473)	**<0.001**

Method 4 The absolute value of dd-cfDNA quantified by the non-pre-amplified dd-PCR (n = 218)					
	n	No	n	Yes	*p*-value
		Median (IQR)		Median (IQR)	
Viral infection	125	0.120 (0.000–0.260)	93	0.200 (0.060–0.475)	**0.034**
Bacterial infection	200	0.163 (0.000–0.390)	18	0.220 (0.058–0.438)	0.518
Fungal infection	207	0.133 (0.000–0.360)	11	0.520 (0.230–0.760)	**0.021**
CLAD	203	0.120 (0.000–0.330)	15	0.470 (0.238–0.840)	**0.001**

Data are presented as median (Md) and interquartile range (IQR). N–number. PCR, Polymerase Chain Reaction; SNP, Single-nucleotide polymorphism; CLAD, Chronic Lung Allograft Disfunction. The statistic calculations were done using Mann-Whitney U test. Significant *p*-values are highlighted in bold.

**FIGURE 2:**
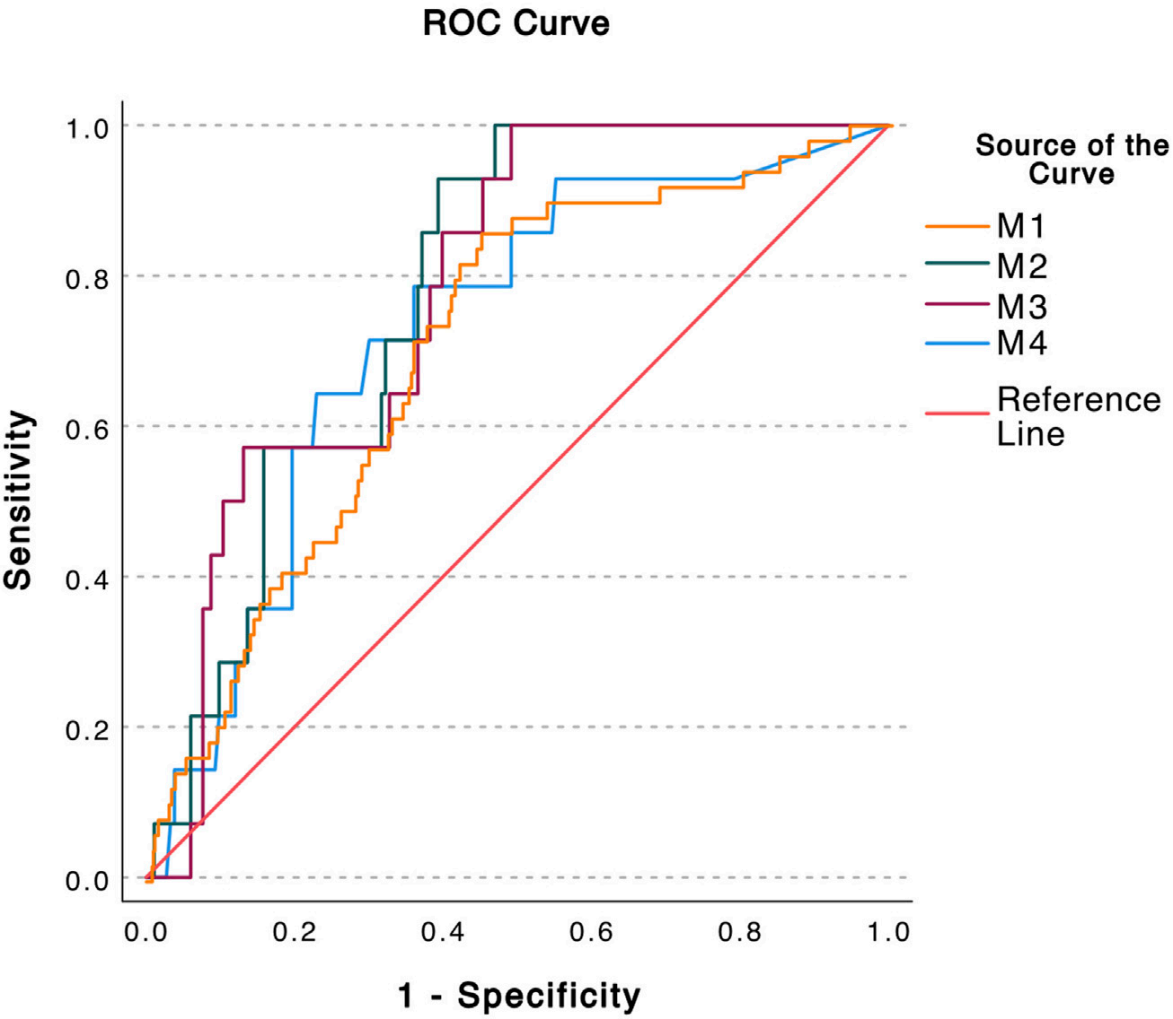
Calculation of the predicative accuracy of the donor fraction (DF) obtained by four different methods by ROC. AUC = Area Under Curve. M1 Method 1 DF calculated from each pre-amplified SNP individually (AUC = 0.709) M2 Method 2 DF calculated from mean of all pre-amplified SNPs per event (AUC = 0.780) M3 Method 3 DF Calculated from the first non-pre-amplified SNP (AUC = 0.778) M4 Method 4 The absolute value of dd-cfDNA quantified by the non-pre-amplified dd-PCR (AUC = 0.726).

ROC analyses by plotting sensitivity versus (1-sensitivity) for analysis of predictive accuracy for all methods are displayed in Figure 2. The AUC for M1= 0.709, for M2 AUC = 0.780, for M3 AUC =0.778 and for M4 AUC = 0.726.

## Discussion

cfDNA as a prediction tool and possible biomarker for rejection after lung transplant was introduced in 2015 [22]. Most studies published have been analyzing the risk of acute rejection, antibody mediated rejection, or undefined rejection [33]. Only a few analyzed the risk of CLAD [41]. In the current study, we found that both DF and absolute levels of dd-cfDNA were significantly higher for CLAD samples than non-CLAD samples. For three of the analyzing methods, there were no statistical differences in test values between the presence and absence of any other clinical events included in the study. For the fourth method, using the absolute value of dd-cfDNA, both viral and fungal infections also had significantly higher values. Furthermore, the ROC AUC values show a fair ability of all methods to discriminate between CLAD and non-CLAD samples. The findings contribute to the pool of evidence for cfDNA as a useful biomarker for CLAD.

It has previously been shown that males generally have higher levels of cfDNA compared to females [42]. Surprisingly, in our series, we found that DF was higher among female than among male patients. Previous studies within the field of heart transplantation have not shown any sex difference in DF [43, 44]. To our knowledge this issue has not be studied in lung transplantation recipients and will warrant further studies.

Based on previous research, we expected higher levels of DF in double lung recipients compared to single ones because of donor lung mass [45]. However, we did not find any difference in DF levels regarding transplantation type. Our results were more in concordance with the findings of Kush et al. [46].

Samples collected at 1 month were observed to have an elevation of cfDNA compared to subsequent samples in the proof-of-concept study [26]. This observation was confirmed in the current study. The reason is likely lingering peri-operative injuries to the allograft. This finding suggests that it would be problematic to include samples drawn up to 1 month after LTx in pooled analyses and likely also in upcoming predictive modelling and establishing of a baseline value for dd-cfDNA from future prospective studies. Furthermore, this is a time point when CLAD can never be present due to its definition. However, these samples could possibly be of value for risk stratification post-transplant, as previously published by Agbor Enoch et al. [21].

There was no difference between the RAS and other subtypes of CLAD in our findings and thus we did not separate the subtypes in our analyses. However, the number of RAS patients were very few and the generalizability of this finding is low.

Although elevated levels of cfDNA have been found at the time points of viral [47] and other microbial [48] infections in transplanted patients, we did not find such an association with DF. We expected a slightly elevated DF in the infection suffers since plasma dd-cfDNA represents the allograft tissue injury. Our results were more in line with studies of Khush et al. [46] and Ju et al. [49], who did not find a significant difference in the plasma dd-cfDNA level between samples gathered with or without infectious events. One possibility is that allograft infection was not defined as only a confirmed serious invasive infection but also included milder forms of the presence of bacterial DNA in the airways with some clinical impact. This heterogeneity probably explains the lack of consistent elevated dd-cfDNA levels that would hypothetically be present in tissue injury [46]. Another possibility, when using DF, is that the inflammatory effect of infection is not isolated to the allograft leading to non-elevated quotas. When comparing the absolute levels of dd-cfDNA of bacterial infections, we see significantly higher values for viral infections which are disseminated, as well as for fungal infections, confirmed with directed bronchoscopy. This is a finding supporting these hypotheses, however it also introduces these conditions as confounders for CLAD.

The results of ROC analyses used to assess the sensitivity and specificity of DF to detect ongoing CLAD were 0.71–0.78, which may be considered acceptable in this context. Similar levels have been obtained in the study using the target-specific amplified ddPCR tests [43]. The AUC value is kept down by false negatives, which could be a result of the samples in the study not being collected in tubes that were not optimized for cfDNA extraction. However, it is not impossible that systemic affliction, in combination with CLAD, provided false low DFs. The sensitivity may be improved by further development of the methodology and using a standardized blood sample collection. Determining cut-off values would perhaps be possible in a further diagnostic system but beyond the scope of the current study. The best sensitivity was shown using M2, but M3 without pre-amplification performed almost as well and is a much faster method. There have been issues concerning the evaluation of cfDNA in clinical practice [27] in part due to different and complex technical approaches, with different efficiencies in testing cfDNA. Using several averaged assays can alleviate this issue. The slightly improved AUC for CLAD discrimination when using averaged assay values compared to using multiple singulars provides some support for this assumption.

Interestingly a DF level ≥1% has been proven as a clinically relevant threshold [21, 22] for cfDNA and graft injury. In this study, even lower levels could be associated with CLAD. The difference is most likely due to higher levels of recipient genomic DNA in the samples in the current data set [50], which, in turn, is caused by preanalytical factors such as degree of hemolysis in collection tubes, transport times, and centrifugation procedures. However, the diverging methodologies preclude any definite conclusions from comparisons of absolute rd-cfDNA levels between studies.

The study is unique in using many long-term stored frozen samples for the detection of cfDNA in lung transplantation. Although all the samples used in the study had been frozen for more than 5 years in ordinary cryo-tubes and no cell-free DNA collection tubes had been used for blood sampling, the method still showed a remarkable quality of the samples. This suggests that secondary site sampling and freezing are possible, which would expand the options for sampling and storage of cfDNA. The rather complicated method can be set up in a limited number of laboratories to cover several transplant programs. However, it is very plausible that using standard sampling equipment and procedures would have rendered fewer negative samples.

The original study was performed several years ago, and follow‐up routines and dominant immunosuppressive regimens have changed since. Furthermore, the collection of serum samples was not performed according to the protocol initially designed for the method [29]. Therefore, the results of this study must be interpreted with caution, awaiting further prospective studies using standardized sampling protocols. Also, no cases of antibody-mediated rejection were found when testing was prompted. However, at the time, no surveillance testing of anti-HLA antibodies was performed. Thus, no data on the effects of anti-HLA antibody dynamics in correlation to cfDNA dynamics was possible to extract.

The major strengths of the study include the long follow-up period, the standardized way in which the surveillance program was performed and how the collection of tests have been carried out and fairly high number of analyzed samples.

Future studies of the current method for cfDNA analysis in lung transplant patients need to be prospective with larger cohorts designed with the purpose of determining practical cut-off values for clinical application. For instance, this study was designed and initiated before the ISHLT consensus document for the standardization of definitions of infections in cardiothoracic transplant recipients [51]. However, infections in our study were deemed clinically relevant in the presence of microorganisms in the airways and assessed by an experienced clinician as clinically relevant. Given the retrospective nature of the available data and our selection inclusion, neither CRP nor anti HLA-antibodies were prospectively collected, and this would be of great interest in future prospective settings. Furthermore, future studies need to define inter‐patient variability and include to analysis of cfDNA response to different types of CLAD as well as dynamics of cfDNA after CLAD has been developed. The analysis of more clinical variables, for examples donor specific antibodies would be of great interest.

In conclusion, in this study we used combined methods for detecting and quantifying both dd‐cfDNA and rd‐cfDNA. We found that, regardless of the method to quantify DF, elevated levels of dd-cfDNA were associated with CLAD development. Further prospective research is warranted to validate the measurement of cfDNA, to predict and avoid complications in a clinical setting.

## Data Availability

The raw data supporting the conclusions of this article will be made available by the authors, without undue reservation.
