# Supplementary material for: Donor Fractions of Cell-Free DNA Are Elevated During CLAD But Not During Infectious Complications After Lung Transplantation
Source: Transpl Int. 2024 Jul 24;37:12772. doi: 10.3389/ti.2024.12772 (PMC11303165; doi:10.3389/ti.2024.12772)
Supplement: Supplementary file 1 [file DataSheet1.zip › Supplementary data.docx]

Supplementary Table S1. Baseline characteristics grouped on sex and CLAD during follow-up.

| **Variable** |  |  |  |  |
| --- | --- | --- | --- | --- |
| **Sex** |  | **Female (n=15)** | **Male (n=11)** | p-value |
| Age at time of transplantation, years | Median (IQR) | 50.0 (40.0 – 64.0) | 52.0 (46.0 – 57.0) | 0.683^a^ |
|  |  |  |  |  |
| BMI (kg/m^2^) | Median (IQR) | 23.1 (19.5 – 28.7) | 23.1 (21.4 – 29.7) | 0.479^a^ |
|  |  |  |  |  |
| Indication for transplantation | Pulmonal fibrosis, n (%) | 2 (18.2%) | 6 (40.0%) | 0.552 |
|  | COPD, n (%) | 6 (54.5%) | 6 (40.0%) |  |
|  | Alpha-1 trypsin deficiency, n (%) | 2 (18.2%) | 1 (6.7%) |  |
|  | Other, n (%) | 1 (9.1%) | 2 (13.3%) |  |
|  |  |  |  |  |
| Type of transplantation | Single, n (%) | 4 (36.4%) | 4 (26.7%) | 0.457 |
|  | Double, n (%) | 7 (63.6%) | 11 (73.3%) |  |
| Mismatch | CMV, n (%) | 4 (26.7%) | 2 (18.4%) | 0.491 ^b^ |
|  | EBV, n (%) | 1 (6.75) | 0 (0.0%) | 0.577 ^b^ |
|  |  |  |  |  |
| **CLAD during follow-up** |  | **No (n=17)** | **Yes (n=9)** |  |
| Female, n (%) |  | 10 (66.7%) | 5 (33.3%) | 0.598 |
| Male, n (%) |  | 7 (63.6%) | 4 (36.4%) |  |
|  |  |  |  |  |
| Age at time of transplantation, years | Median (IQR) | 50.0 (40.5 – 58.0) | 56.0 (46.5 – 70.0) | 0.148^a^ |
|  |  |  |  |  |
| BMI (kg/m^2^) | Median (IQR) | 22.4 (18.4 – 26.9 | 24.0 (21.8 – 29.5) | 0.274^a^ |
|  |  |  |  |  |
| Indication för transplantation | Pulmonal fibrosis, n (%) | 6 (35.3%) | 2 (22.2%) | 0.361 |
|  | COPD, n (%) | 7 (41.2%) | 5 (56.6%) |  |
|  | Alpha-1 trypsin deficiency, n (%) | 1 (5.9%) | 2 (22.2%) |  |
|  | Other, n (%) | 3 (17.6%) | 0 (0.0%) |  |
|  |  |  |  |  |
| Type of transplantation | Single, n (%) | 4 (23.5%) | 13 (76.5%) | 0.255 |
|  | Double, n (%) | 4 (44.4%) | 5 (55.6%) |  |
|  |  |  |  |  |
| Mismatch | CMV, n (%) | 4 (23.5%) | 2 (22.2%) | 0.668 ^b^ |
|  | EBV, n (%) | 1 (5.9%) | 0 (0.0%) | 0.654 ^b^ |

n – number. IQR – interquartile range. BMI – Body Mass Index. COPD – chronic obstructive pulmonary disease. Mismatch – seropositive donor and seronegative recipient. CLAD, chronic lung allograft dysfunction. CMV – Cytomegalovirus. EBV – Epstein-Barr virus. The statistic calculations were done using Chi-Square test with exception for ^a^ – Mann-Whitney U test and ^b^ – Fisher’s Exact Test.

Supplementary Table S2 First-Month vs Later than first-month samples.

| **Variable** | **Median (IQR)** | **Variable** | **Median (IQR)** | **p- value** |
| --- | --- | --- | --- | --- |
| **First Month** |  | **Later** |  |  |
| M 1 | 0.248 (0.083-0.472) | M 1 | 0.056 (0.021-0.154) | **<0.001** |
| M 2 | 0.223 (0.093-0.423) | M 2 | 0.060 (0.025-0.151) | **<0.001** |
| M 3 | 0.197 (0.094-0.322) | M 3 | 0.070 (0.152-0.185) | **0.005** |
| M 4 | 0.399 (0.109-1.165) | M 4 | 0.168 (0.000-0.403) | **0.007** |

M1 Method 1 DF calculated from each amplified SNP individually.

M2 Method 2 DF calculated from mean of all amplified SNP’s per event.

M3 Method 3 DF Calculated from the first unamplified SNP.

M4 Method 4 The absolute value of dd-cfDNA quantified by the unamplified dd-PCR.

Data are presented as median and interquartile range (IQR). The statistic calculations were done using Mann-Whitney U test. Significant p-values are highlighted in bold.
